# Impact of Different Synchrotron Flattop Operation Modes on 4D Dosimetric Uncertainties for Scanned Carbon-Ion Beam Delivery

**DOI:** 10.3389/fonc.2022.806742

**Published:** 2022-02-11

**Authors:** Pengbo He, Qiang Li

**Affiliations:** ^1^ Institute of Modern Physics, Chinese Academy of Sciences, Lanzhou, China; ^2^ Key Laboratory of Heavy Ion Radiation Biology and Medicine, Chinese Academy of Sciences, Lanzhou, China; ^3^ Key Laboratory of Basic Research on Heavy Ion Radiation Application in Medicine, Gansu Province, Lanzhou, China; ^4^ University of Chinese Academy of Sciences, Beijing, China

**Keywords:** scanned carbon-ion therapy, moving target, synchrotron flattop operation, motion mitigation, 4D dose calculation

## Abstract

**Purpose:**

The characteristic of pulsed beam delivery for synchrotron-based carbon-ion radiotherapy has led to the emergence of many scanning scenarios in order to improve the treatment efficiency and accuracy of moving target volume. Here, we aim to evaluate a novel breathing guidance motion mitigation performance under different synchrotron flattop operation modes in carbon-ion radiotherapy.

**Methods:**

With the use of twelve 4DCT datasets of lung cancer patients who had been treated with respiratory-gated carbon-ion pencil beam therapy, range-adapted internal target volume (raITV) plans were optimized. Under the fixed flattop with single-energy and extended flattop with multi-energy synchrotron operation modes, the 4D treatments with breathing guidance and free breathing-based gated phase-controlled rescanning (PCR) beam delivery were simulated. Dose metrics (D95 and D5–D95 in clinical target volume (CTV)) and treatment time of the resulting 4D plans were compared.

**Results:**

The two synchrotron operation modes provided different scanning dynamics. For the free breathing-based PCR method delivered in the extended flattop operation mode, the averaged CTV-D95 values were 90.4% ± 3.7%, 95.4% ± 1.7%, 96.9% ± 1.5%, 97.2% ± 1.5%, and 97.3% ± 1.5% for the 1-scanning, 2-PCR, 4-PCR, 6-PCR, and 8-PCR, respectively. For the breathing guidance-based PCR method delivered in the extended flattop mode, these values were 89.1% ± 4.0%, 97.0% ± 1.4%, 98.2% ± 0.7%, 98.6% ± 0.7%, and 98.9% ± 0.7%, respectively. However, CTV-D95 significantly increased to 98.5% ± 1.0% even with just 1-scanning breathing guidance-based fixed flattop operation mode (p < 0.01). Moreover, there was no significant difference in treatment time among the three technical combinations (p > 0.15).

**Conclusions:**

The combination of the breathing guidance and PCR methods should be an alternative way for motion mitigation for the fixed flattop synchrotron operation mode. The target dose coverage and homogeneity could be further improved by the combination of the breathing guidance and PCR methods than the traditional PCR-only technology for the extended flattop synchrotron operation mode.

## Introduction

For the scanning beam delivery, target motion could induce distorted dose distributions within the target volume and cause serious overdose irradiation to the surrounding normal tissues (1–4). Theoretically, tracking (5, 6) should be the most accurate and efficient method for motion management. However, due to the real-time 3D imaging limitation for lateral spot position and beam range variation compensation, it has not been applied clinically at present. The combination of gating (7, 8) and rescanning (9–13) methods has been widely used for target motion compensation. With the gating method, the residual target motion could be reduced to be less than 6 mm within the gating window (GW), and then the rescanning method could be further used to compensate the residual target motion-induced dose distortion (14). For carbon-ion beam radiotherapy, the synchrotron magnetic excitation cycle (MEC) generally includes five operation phases: acceleration phase, flattop phase (beam extraction), re-acceleration phase (acceleration to constant energy to avoid hysteresis), deceleration phase, and de-well time. The target volume could only be irradiated when the gate-on phase is coincidental with the beam extraction phase, and the effectiveness of treatment is generally low (15, 16).

**Figure 1:**
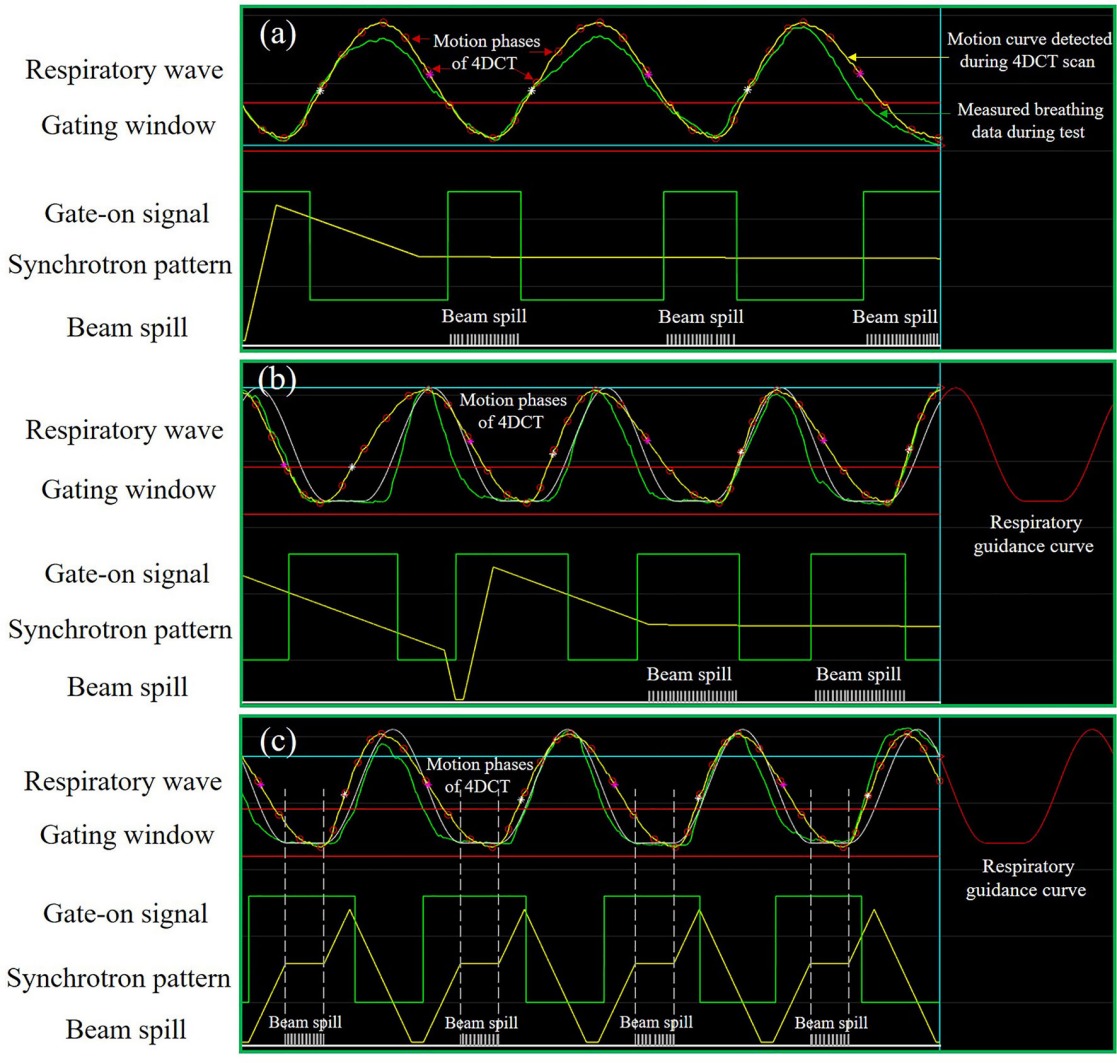
Schematic diagram of the synchrotron operation modes and motion mitigation methods. **(A)** Free breathing-based gated phase-controlled rescanning (PCR) method delivered under the extended flattop synchrotron operation. **(B)** Breathing guidance-based gated PCR method delivered under the extended flattop synchrotron operation. **(C)** Breathing guidance method delivered under the fixed flattop synchrotron operation.

Extended synchrotron flattop operation mode developed by the National Institute of Radiological Sciences (NIRS), Japan, was used to increase the treatment efficiency of gated beam delivery (9, 14, 17–20). As shown in **Figures 1A, B**, the synchrotron flattop is extended, which means that the available beam extraction time is increased according to the dose requirement of a specific treatment plan, and when the respiration gate is open, the beam is extracted using the radiofrequency (RF)-knockout method. Therefore, the respiratory gate-on signal is always synchronized with the beam extraction phase, and thus the beam delivery efficiency is expected to be high. Additionally, the multiple-energy operation mode is employed to quickly change the beam energy within 0.3 s to realize fast raster scanning irradiation (19). With the extended flattop of the multi-energy operation pattern, carbon ions are initially accelerated to the maximum energy and then decelerated to the lower desired energies as provided by the treatment plans (17–20). The application of the extended flattop operation at the desired energy to extract the beam is shown in **Figures 1A, B**. The layered phase-controlled rescanning (PCR) method is realized by adjusting the beam current level to realize the match between the GW time and the beam delivery time for each energy slice (19, 21). A combination of the above-described technologies could compensate for the residual target motion within the GW.

Another solution is the audio-visual biofeedback (AV-BFB) respiratory guidance method used in the traditional synchrotron operation pattern, e.g., fixed flattop with a single-energy operation (22, 23). The carbon ions are accelerated to the desired energy directly, and then the beam is extracted during the synchrotron flattop phase. The beam energy remains constant throughout the beam extraction phase until the end of the flattop phase, and then the next energy beam is accelerated as required by the treatment planning steering file. Based on the average breathing period and amplitude, the patient-specific respiratory guidance curve with a short breath-hold (BH) phase could be established and displayed on a screen in front of the patient. The de-well time between the last deceleration phase and the next acceleration phase of the MEC could be properly adjusted to synchronize the patterns between the synchrotron and breathing. Under the instruction of the breathing guidance curve, the patient breathing hold phases could be synchronized with the synchrotron flattop phases, as shown in **Figure 1C**, such that the target volume is irradiated during the BH phases and remains nearly static for every breathing cycle. Thus, both the treatment efficiency and precision are expected to be improved against the traditional gating method.

The above two synchrotron operation modes provide different scanning dynamics. A sequence of scanning points needs to be scanned across the target volume point by point and slice by slice to achieve a well-defined target dose distribution. In the direction perpendicular to the beam, for a given energy slice, the beam current is different between the fixed flattop operation and the extended flattop operation, which depends on the GW time. In the beam direction, for different energy slice scanning, a fast energy change within 0.3 s could be achieved for the multiple-energy operation, while a fixed energy change time of ≈3 s is needed for the single-energy operation (19, 22). Thus, the delivery dynamics of the exact timing of each scanning spot is quite different for the extended flattop and fixed flattop synchrotron operations. For moving target irradiation, any variation in the beam delivery timeline could change the interplay effect between the target motion and beam delivery process and result in different 4D dose distributions (4DDDs) (24). However, due to the synchronization patterns between the synchrotron MEC and respiratory period, the BH phases are always coincidental with the synchrotron beam extraction phases for every breathing cycle. Theoretically, the treatment efficiency should be similar for the PCR and AV-BFB respiratory guidance methods.

To our knowledge, there is no study comparing the motion compensation effects of PCR and AV-BFB respiratory guidance as a function of different synchrotron flattop operation modes. Furthermore, the effectiveness of AV-BFB performed on the extended flattop with multi-energy (EFME) synchrotron operation has never been investigated before. It is valuable to compare the performances of these therapeutic techniques in order to make a decision for synchrotron-based carbon-ion beam treatment for moving targets. This study focused on the systematical evaluation of potential differences in both the treatment efficiency and 4DDD of the different technique combinations above, taking into account different beam delivery time sequences (BDS).

## Materials and Methods

### Motion Data and Treatment Planning

A retrospective analysis was conducted for 12 randomly selected lung cancer patients (upper lung lobe, 2; middle lung lobe, 7; and lower lung lobe, 3) using scanning carbon-ion beam treatment at the Shanghai Proton and Heavy Ion Center (SPHIC), China. All data used in this study were approved by the institutional research ethics committee of the Institute of Modern Physics, Chinese Academy of Sciences. The requirement for patient consent was waived with the approval of the ethics committee given that patient anonymity was ensured. 4DCT datasets for the patients were acquired with the SOMATOM Definition AS 64 multi-slice CT scanner. During the 4DCT scanning, the respiration signals were detected with the AZ-733V respiratory gating system. Ten equally spaced bins 4DCT datasets were reconstructed (T00, peak inhalation; T50, around peak exhalation).

Irregular breathing-induced 4DDDs during the whole simulated treatment process were evaluated with 12 sets of the respiratory data, which were selected in a way where the breathing curves had motion amplitudes similar to those of the target volumes in the 4DCTs. All the breathing data are long enough to cover the entire beam delivery course under the free-breathing and respiratory guidance maneuvers acquired in previous studies (22, 23). The average motion magnitude was 7.3 ± 2.6 mm with a mean (range) period of 4.3 (3.4–5.6) s calculated using the Fourier analysis for the motion data.

The gross tumor volumes (GTVs) and organs at risk (OARs) were contoured by experienced radiation oncologists on T50. A uniform 5-mm margin was added to the GTVs to form the clinical target volumes (CTVs). Then translating the 4DCT images and volume of interest (VOI) data as DICOM files to a custom-built 4Dtool, the VOI data were propagated from the reference phase to the other phases using the B-spline deformable registration (25, 26). The beam range changes caused by respiratory motion were properly associated with designing range-adapted internal target volume (raITV) (27). The minimum and maximum water equivalent path length (WEPL) values were calculated from the beam entrance to each voxel within the CTV at each motion phase (T40–T60) along a ray-line using a ray-casting algorithm. Then two points on the reference phase (T50) with the same minimum and maximum WEPL values were calculated on the same ray-line, and then ±2 mm-WEPL range uncertainty margins were added in the proximal and distal sides. The raITV could be constructed for this beam field by repeating the above calculation process for every voxel within the CTV on all rays. A pair of horizontal and vertical orthogonal beams perpendicular to the superior-to-inferior direction of the patients from the ipsilateral side of the tumor was designed. The single-field uniform dose (SFUD) optimization with a prescription dose of 2 Gy (RBE) under a single fraction was performed on T50 for each plan in the matRad (28). The scan point and energy layer spacings were set to 3- and 3-mm WEPL, respectively. After the optimization, beam delivery steering files (including scan point positions, energy list, and optimized spot weights) were exported for the subsequent 4D dynamic BDS calculations.

### Beam Delivery Simulation System

To simulate the beam delivery processes under different synchrotron operation patterns, we developed a homemade beam delivery simulation (BDsim) system to simulate the different accelerator operation modes of extended flattop and fixed flattop. In this system, the beam delivery parameters such as beam intensity, total particle number per pulse, and the lateral scanning speeds in the x and y directions at the iso-centric plane could be set manually. The multiple-energy and single-energy accelerator operation modes were established based on the magnetic excitation curves of the Heavy Ion Medial Accelerator in Chiba (HIMAC) in Japan and Heavy Ion Medical Machine (HIMM) in China, respectively (19, 20, 23).

**Figure 2:**
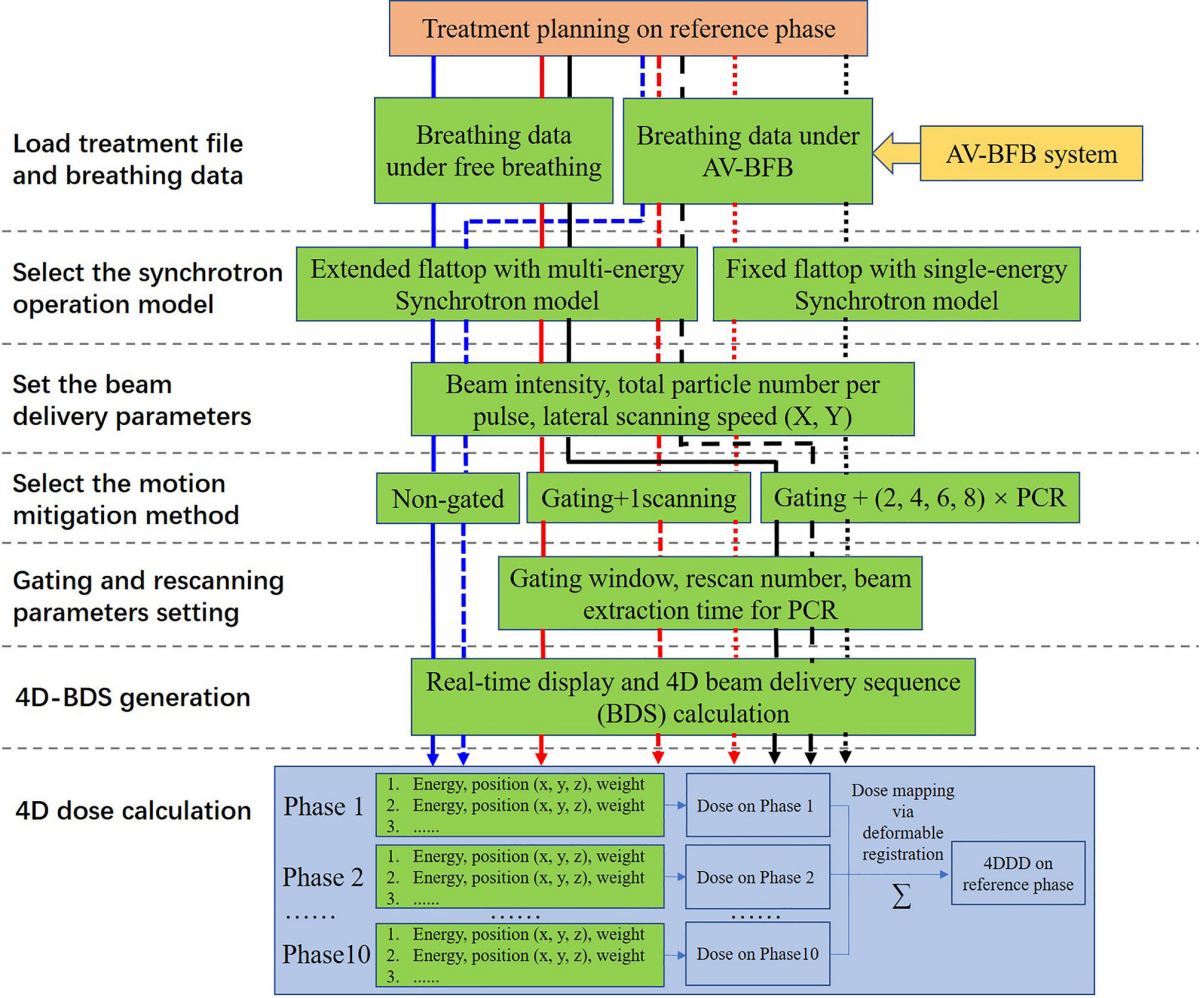
Schematic representation of the simulation strategy and calculation process adopted in this study.

As shown in **Figure 2**, after loading the breathing data and the treatment steering file to the BDsim, the specific accelerator operation mode could be selected, and the corresponding beam delivery parameters could be set. Any combinations of the motion mitigation methods such as respiratory gating, rescanning with or without PCR, and AV-BFB respiratory guidance are available. After the duty cycle (DC) value, the rescanning number, and the beam extraction time for each breathing cycle for the PCR method were set, the treatment simulation process could be started with or without respiratory guidance. As shown in **Figure 1**, the motion signals such as breathing data and the motion curve detected during the 4DCT scan, the gating parameters (threshold and GW), the respiratory guidance curve, and the synchrotron pattern could be displayed on the BDsim system in real time to show the beam delivery process. The repeated display of the motion curve detected during the 4DCT scan was used for real-time motion phase calculation as shown in **Figure 1** for the labeled motion phases of 4DCT. For the current scan point, the motion phase on the motion curve detected during the 4DCT scan was calculated firstly and denoted as phase A. In order to consider the irregular motion-induced spot positions’ shift, the offset between the positions of the current breathing curve loaded from the detected free breathing or breathing guidance tests and phase A was calculated secondly. If the motion curves detected during the 4DCT scans and the treatments coincided perfectly, the offset would be zero. Otherwise, the two orthogonal positions of the current spot perpendicular to the beam direction on phase A were calculated by adding the offset to the original planning spot positions. The dose weight of the current spot on phase A is determined by the beam intensity, scanning speed, and movement velocity of the target volume. Therefore, the sub-spot positions between the two continuous scanning spots were also calculated, as the raster scanning beam delivery method was used while the beam was not interrupted during the spot shift. In combination with the sequential spot beam delivery process, the distributions of local spot positions and corresponding spot weights on each motion phase for all scan points could be determined. The irregular breathing-induced dose distortion could be properly associated with this way. The redistributed 4D steering file was used for the subsequent 4D dose calculations (4DDCs).

### 4D Dose Calculations

After the synchrotron operation mode is properly selected, the horizontal scanning speeds, vertical scanning speeds, and total particle number per pulse were set to 100 mm/ms, 50 mm/ms, and 4.0×10^8^ ppp, respectively. The beam intensity was a variable quantity depending on the optimized total particle number of each energy slice and the DC value of the GW. The moving phases’ sub-dose distributions were calculated separately in the matRad (28) with the local spot positions and weight distributions of the generated 4D steering files from the BDsim system and then transferred to the custom-built 4Dtool. Based on the positional relationships between voxels at T50 and moving phases established during the contour propagation process, the sub-dose distributions at moving phases could be transformed to T50 directly and summed up to form the final 4DDD (29).

### Assessment on Treatment Efficiency and Dosimetric Parameters

All the experiment combinations and calculation processes are shown in **Figure 2**. For the free-breathing conditions, the 4DDDs were only calculated under the extended flattop synchrotron operation pattern as shown in **Figure 1A**, because under the fixed flattop mode, the free-breathing GW could not always synchronize with the synchrotron flattop, resulting in increased residual target motion and low treatment efficiency (20, 21). For the breathing guidance maneuvers, the 4DDDs were evaluated for both the extended flattop (shown in **Figure 1B**) and fixed flattop (shown in **Figure 1C**) synchrotron modes. The dose deliveries without rescanning (once scanning) and with rescanning of 2×, 4×, 6×, and 8× layered PCR were simulated with the GW of 30% DC for all the technical combinations described above. Therefore, a total of 204 4D scenarios were analyzed including variations in patient geometry (12×), free-breathing and breathing guidance-based gating, and PCR delivery (non-gated and gated with 1-scanning, 2-PCR, 4-PCR, 6-PCR, and 8-PCR) under the extended flattop synchrotron operation, and breathing guidance maneuvers (1-scanning, 2-PCR, 4-PCR, 6-PCR, and 8-PCR) under the fixed flattop mode.

The qualities of the resulting 4DDDs were compared using the dose homogeneity index (HI) (D5 − D95)/D_presc_ and percentage dose coverage of 95% (D95) from the dose–volume histograms (DVHs) of the CTV. The predicted treatment time as a function of the synchrotron pattern and beam delivery scenario was also analyzed. Both the D95 and HI values were averaged over all patients, and the corresponding SDs were calculated. Statistical significance of differences in dose assessment metrics and treatment time was examined using the Wilcoxon signed-rank test, and p < 0.01 was considered statistically significant.

## Results

### Comparison of the Beam Delivery Time Sequences for All Scenarios

**Figure 3:**
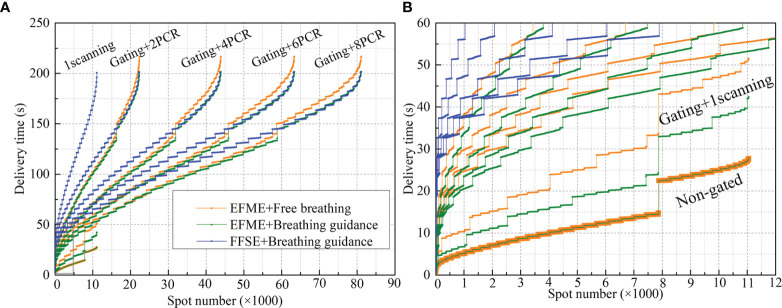
Beam delivery time sequences of free-breathing and breathing guidance plans under different motion mitigation scenarios (non-gated, gating + 1 scanning, gating + phase-controlled rescanning (PCR)) delivered in the extended flattop with multi-energy (EFME) and fixed flattop with single-energy (FFSE) synchrotron operation modes. Global graph with all the delivered spots **(A)** and partially enlarged graph **(B)** show details.

The spot beam delivery time sequences of the same example plan differed significantly as a function of the motion mitigation methods as shown in **Figure 3**, which could change the dynamic interaction process and affect the final 4DDDs directly. For the breathing guidance maneuvers under the fixed flattop synchrotron operation, the steps on the BDS curves indicated the energy changing time and the flat areas represented the scanning points of the corresponding energy layers. A rapid rise of the BDS curves at the first and last few energy layers was presented due to the smaller number of scanning points in the distal and proximal ends of the target volume. As the number of scanning points increased for the intermediate energy layers, the BDS curves became gradually smooth. For the breathing guidance maneuvers under the extended flattop synchrotron operation mode, the BDS curves became smoother due to the fast energy switching time compared to the fixed flattop mode. Additionally, a bigger time step presented on the BDS curves around 140 s for the extended flattop mode represented the beam pulse switching time point, indicating that beam was extracted completely for the current pulse and another synchrotron operation cycle was needed to finish the treatment. The same situation occurred in the BDS curves for the extended flattop synchrotron operation mode under free-breathing conditions. However, a slight increase in delivery time was presented due to the irregularity of the free-breathing curve, and more than one breathing cycle might be needed for an energy slice scanning. According to the BDS curves of 1-time scanning scenarios, the total spot number was around 1.1 × 10^4^. It increased exponentially with the increase of rescanning number, but the total treatment time for the rescanning scenarios remained almost unchanged due to the beam intensity control mechanism of the PCR method.

### Comparison of the Dosimetric Impacts on 4D Dose Distributions

**Figure 4:**
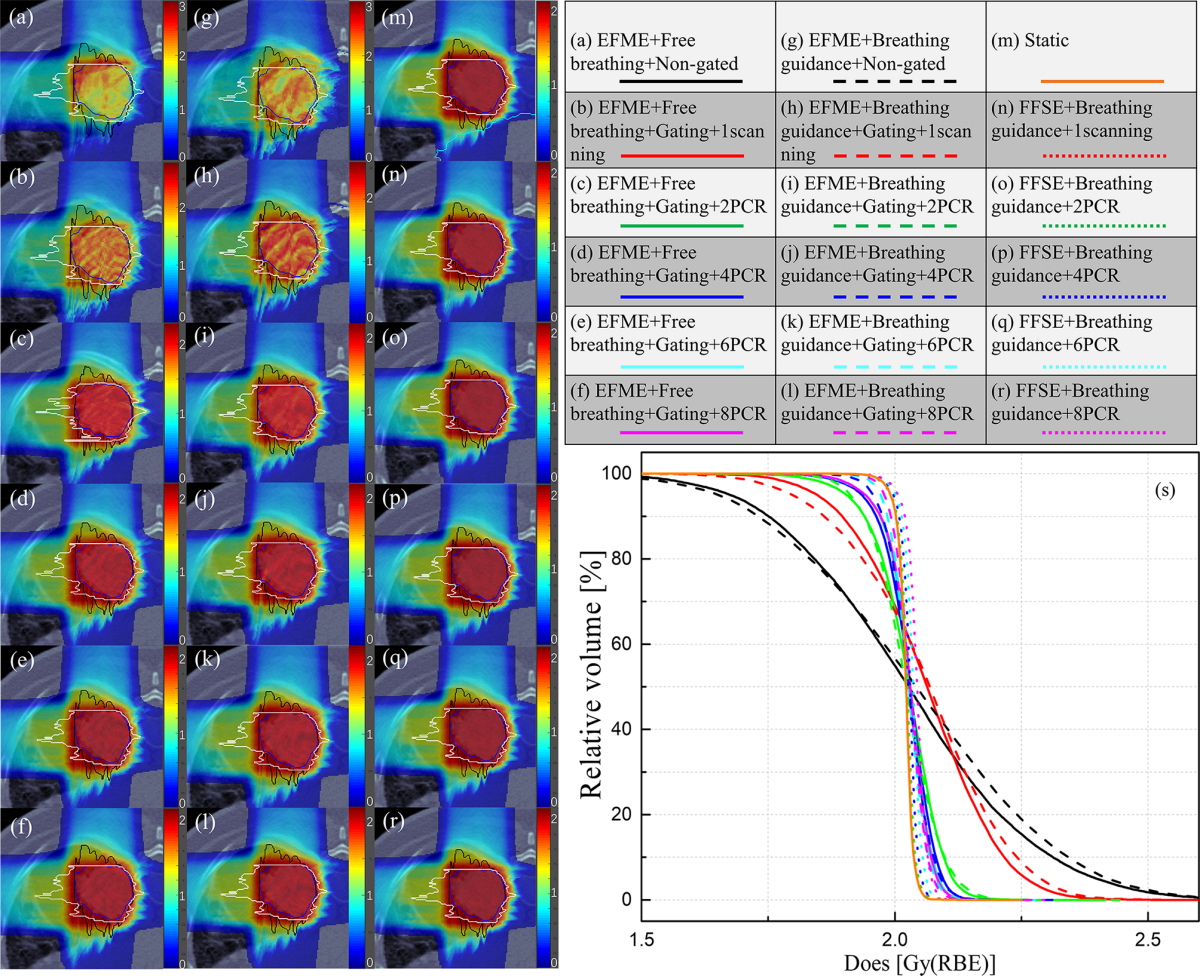
**(A–F)** 4D dose distributions for a lung cancer case under the non-gated free-breathing and gating with phase-controlled rescanning (PCR) methods delivered in the extended flattop with multi-energy (EFME) mode. **(G–L)** Breathing guidance maneuvers in combination with gating and PCR methods under the extended flattop with multi-energy (EFME) operation. **(M)** Static case. **(N–R)** Breathing guidance maneuvers in combination with gating and PCR methods under the fixed flattop with single-energy (FFSE) synchrotron operation mode. **(S)** Corresponding dose–volume histogram bands. Range-adapted internal target volumes (raITVs) are depicted as black and white lines for each irradiation field.

**Figure 4** displays an example lung case of 4DDDs under the static, free-breathing, and breathing guidance conditions in combination with gating and PCR methods delivered under the fixed flattop and extended flattop synchrotron operations. The free-breathing tests were performed in the extended flattop mode, and insufficient CTV dose coverage was observed as shown in **Figure 4A**. Even the 1-time scanning scenario (**Figure 4B**) was compensated with gating, and the dose distribution was also distorted compared to the static case shown in **Figure 4M**. With the combination of gating and PCR methods as shown in **Figures 4C–F**, both the CTV dose coverage and homogeneity became better. The same situation has occurred on the breathing guidance maneuvers under the extended flattop mode as shown in **Figures 4G–L** because the residual motion within the GW during beam delivery could still cause the interplay effects as shown in **Figure 1B**. However, for the breathing guidance maneuvers under the fixed flattop operation, as shown in **Figures 4N–R**, the dose distributions were almost restored to the same as the static situation regardless of rescanning number. **Figure 4S** displays the corresponding dose-volume histogram bands.

**Figure 5:**
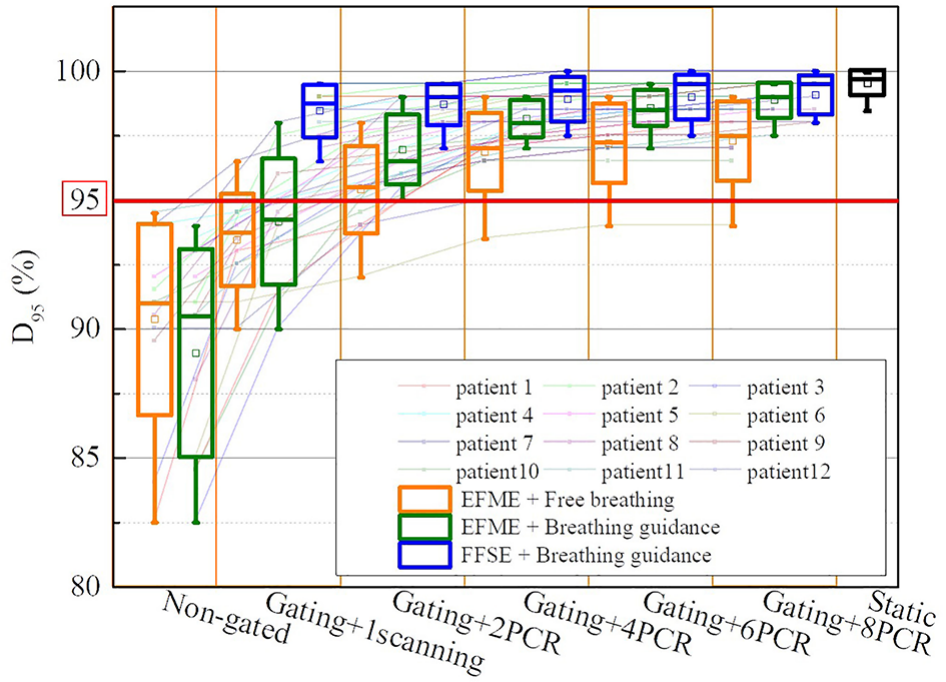
Boxplots of the clinical target volume (CTV) dose coverage D95 over all cases of static, free-breathing, and breathing guidance maneuvers in the combination with gating and phase-controlled rescanning (PCR) methods delivered in the extended flattop with multi-energy (EFME) and fixed flattop with single-energy (FFSE) synchrotron operation modes. The box plots represent one SD, the bands in the boxes are medians, and the extremes are the maximum and minimum values for each dataset.

**Figure 5** displays the dose coverage parameters (CTV-D95) for all lung cancer cases. For the static cases, the average CTV-D95 value was 99.6% ± 0.5%. For the extended flattop synchrotron operation mode, the average CTV-D95 values for the free-breathing and breathing guidance maneuvers without gating and rescanning methods were 90.4% ± 3.7% and 89.1% ± 4.0%, respectively, and slightly increased to 93.5% ± 1.8% and 94.2% ± 2.4% for the 1-time gating scanning, respectively. For the free-breathing maneuvers with the combination of gating and PCR methods, the CTV-D95 values significantly (p < 0.01) increased to 95.4% ± 1.7%, 96.9% ± 1.5%, 97.2% ± 1.5%, and 97.3% ± 1.5% for the 2-PCR, 4-PCR, 6-PCR, and 8-PCR, respectively, and increased to 97.0% ± 1.4%, 98.2% ± 0.7%, 98.6% ± 0.7%, and 98.9% ± 0.7% for the breathing guidance maneuvers, respectively. Therefore, the target dose coverage could be further improved by the breathing guidance method under the extended flattop synchrotron operation mode. However, the CTV-D95 values for the breathing guidance and in combination with the PCR method in the fixed flattop mode were 98.5% ± 1.0%, 98.7% ± 0.8, 98.9% ± 0.9, 99.0% ± 0.9, and 99.1% ± 0.8 for the 1-scanning, 2-PCR, 4-PCR, 6-PCR, and 8-PCR, respectively, indicating that the breathing guidance method could further improve the target dose coverage in the fixed flattop mode (p < 0.01).

**Figure 6:**
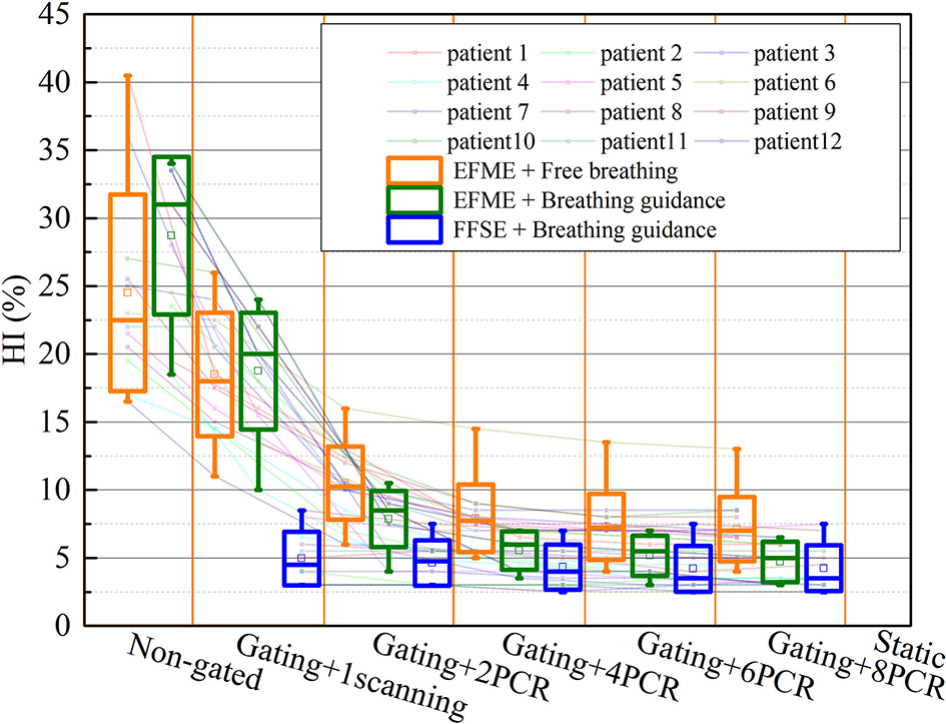
Values of the dose homogeneity index (HI) over all cases of static, free-breathing, and breathing guidance maneuvers in the combination with gating and phase-controlled rescanning (PCR) methods delivered in the extended flattop with multi-energy (EFME) and fixed flattop with single-energy (FFSE) synchrotron operation modes. The box plots represent one SD, the bands in the boxes are medians, and the extremes are the maximum and minimum values for each dataset.

**Figure 6** shows the same trends of dose homogeneity index (HI) distributions as the dose coverage values for all the scenarios. For the static cases, the average HI value was 2.5% ± 0.8%. For the free-breathing and breathing guidance without or with gating in the extended flattop mode, the dose distributions were distorted seriously (HI = 24.5% ± 7.2%, 28.7% ± 5.8%, 18.5% ± 4.5%, and 18.8% ± 4.3%). On the other hand, the HI values gradually decreased to 10.5% ± 2.7%, 7.9% ± 2.5%, 7.3% ± 2.4%, and 7.1% ± 2.4% for the 2-PCR, 4-PCR, 6-PCR, and 8-PCR, respectively, for the free-breathing maneuvers, and decreased to 7.9% ± 2.1%, 5.5% ± 1.4%, 5.2% ± 1.5%, and 4.7% ± 1.5%, respectively, for the breathing guidance maneuvers. However, the dose homogeneities for the breathing guidance maneuvers in the fixed flattop mode were almost kept constant (HI ≈ 4.5%) regardless of rescanning number (p > 0.1) and were significantly improved compared to those of the free-breathing- or breathing guidance-based PCR method in the extended flattop mode (p < 0.01).

### Comparison of the Treatment Efficiencies for Different Motion Mitigation Methods

**Figure 7:**
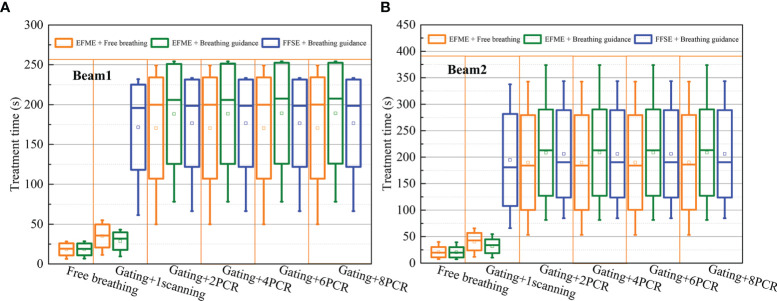
Boxplots of the treatment time over all cases under free-breathing and breathing guidance maneuvers in combination with gating and phase-controlled rescanning (PCR) methods delivered in the extended flattop with multi-energy (EFME) and fixed flattop with single-energy (FFSE) synchrotron operation modes Treatment time distributions for **(A)** beam1 and **(B)** beam2. The box plots represent one SD, the bands in the boxes are medians, and the extremes are the maximum and minimum values for each dataset.

**Figure 7** shows a comparison of the treatment efficiencies of the free-breathing and breathing guidance maneuvers in combination with gating and PCR methods under the fixed flattop and extended flattop synchrotron operations. For the free-breathing and breathing guidance without or with gating maneuvers, the treatment times were only 18.3 ± 7.6, 18.3 ± 7.4, 35.1 ± 14.4, and 28.7 ± 11.0 s, for the beam 1 due to the fast energy switching (≈0.3 s) in the extended flattop mode. However, the average treatment time (≈170.5 ± 63.5 s, 188.3 ± 62.8 s) was almost kept constant (p > 0.06) under the PCR beam deliveries due to the adjustment of beam current level to realize the match between the GW time and the beam delivery time for each energy slice. For the breathing guidance maneuvers in the fixed flattop mode, the average treatment time for 1-scanning was 171.6 ± 53.4 s, while it slightly increased to 176.6 ± 54.8 s for the rescanning maneuvers (p < 0.01) mainly due to the unexpected breathing-induced occasional incomplete irradiation of certain energy slices within the current level-reduced synchrotron pulses of PCR. However, there were no significant differences (p > 0.15) between the three technical combinations (e.g., Fixed flattop with single-energy (FFSE) + Breathing guidance, EFME + Breathing guidance, and EFME + Free-breathing modes), and beam 2 showed the same trend.

## Discussion

A comprehensive 4DDD investigation was performed in this study to compare the dosimetric influence of synchrotron operation modes and motion mitigation techniques on 4DDD and treatment delivery time. The differences in beam delivery time sequence, target dose coverage, dose homogeneity, and treatment efficiency have been evaluated and compared for the breathing guidance and free breathing-based PCR maneuvers delivered in two different synchrotron operation modes. The results indicate that the PCR method is an effective way for dose distortion compensation caused by target motion. The CTV dose coverage and homogeneity could be further improved in the combination of breathing guidance technology under both the fixed flattop and extended flattop synchrotron operations, while no significant difference in treatment time was observed.

For the fixed flattop mode with the breathing guidance maneuvers, the BH phases at the end of the guidance curves were designed to synchronize with the synchrotron beam extraction phases. Therefore, the residual target motion within the GW could be minimized in contrast to the free-breathing and breathing guidance maneuvers under the extended flattop mode, where larger residual motion-induced interplay effects within the GW could distort the dose distributions. This may explain the phenomenon that there were no significant differences in both the CTV dose coverage and homogeneity using either 1-scanning or 8 PCRs under the fixed flattop mode with breathing guidance maneuvers. But they were significantly improved for the free-breathing and breathing guidance-based PCR maneuvers under the extended flattop mode. However, even in the extended flattop operation mode, the dosimetric parameters were much improved with the breathing guidance compared to the free-breathing maneuvers, because of the increased repeatability of the respiratory waveform and thus the accuracy of beam delivery. Additionally, the PCR method was used by adjusting the beam current level to realize the match between the GW time and the beam delivery time for each energy slice. Only one energy slice could be scanned per respiratory cycle regardless of fast energy switching of the extended flattop operation mode, which coincides with the pulsed energy changing pattern of the fixed flattop mode. Therefore, no difference in treatment efficiency was observed between the breathing guidance and free breathing-based PCR maneuvers delivered under the fixed flattop and extended flattop modes.

One limitation of this study is that the lung cancer patients’ 4DCT datasets were used for 4DDCs, but the motion data of healthy volunteers were acquired for different breathing maneuvers (22, 23). In order to focus on the irregular motion-induced dose distortion, the motion data during the whole beam delivery process should be considered in the 4DDCs, but the current imaging system could not realize real-time 3DCT scan. Therefore, in this study, an alternative way is to select 4DCT datasets with similar motion patterns with the breathing curves for 4DDCs. In order to reconstruct the 4DDDs in the treatment process more accurately, the 4DCT dataset should be obtained in real time. A possible approach is to collect 4D-MRI data in real time and convert them into 4DCT data (30). Another promising approach is to collect real-time DR images during treatment and translate them to real-time 4DCT data by the artificial intelligence (AI) method (31). These techniques remain to be further investigated.

## Conclusions

Gating in combination with the PCR method could increase the dose distribution qualities effectively for scanning carbon-ion treatment in the extended flattop synchrotron operation mode. However, the CTV dose coverage and homogeneity could be further improved in combination with breathing guidance technology under both the fixed flattop and extended flattop synchrotron operation modes. There was no significant change in treatment efficiency with the involvement of the guidance method. Therefore, if only the fixed flattop operation mode is available for one treatment center, the combination of the breathing guidance and PCR methods should be an alternative way for target motion management. If the more advanced extended flattop operation mode is equipped, the combination of the breathing guidance and PCR methods could further improve the treatment precision than the PCR-only one.

## Data Availability Statement

The raw data supporting the conclusions of this article will be made available by the authors, without undue reservation.

## Ethics Statement

The studies involving human participants were reviewed and approved by the institutional research ethics committee of the Institute of Modern Physics, Chinese Academy of Sciences. The ethics committee waived the requirement of written informed consent for participation.

## Author Contributions

PH and QL devised the project. PH developed the BDsim and 4Dtool, and QL supervised this work. PH performed the simulation study and gathered data. PH and QL contributed to analyzing the data of this study. The original draft was written by PH and reviewed by QL. All authors contributed to the article and approved the submitted version.

## Conflict of Interest

The authors declare that the research was conducted in the absence of any commercial or financial relationships that could be construed as a potential conflict of interest.

## Publisher’s Note

All claims expressed in this article are solely those of the authors and do not necessarily represent those of their affiliated organizations, or those of the publisher, the editors and the reviewers. Any product that may be evaluated in this article, or claim that may be made by its manufacturer, is not guaranteed or endorsed by the publisher.

## Supplementary Material

Supplementary Video 1 The combination of gating with phase-controlled rescanning methods in the fixed flattop with single-energy synchrotron operation mode under the breathing guidance maneuver.

Supplementary Video 2 The combination of gating with phase-controlled rescanning methods in the extended flattop with multi-energy synchrotron operation mode under the free breathing maneuver.

## References

[1] Phillips MH Pedroni E Blattmann H Boehringer T Coray A Scheib S. Effects of Respiratory Motion on Dose Uniformity With a Charged Particle Scanning Method. Phys Med Biol (1992) 37(1):223–34. doi: 10.1088/0031-9155/37/1/016 1311106

[2] Bert C Grozinger SO Rietzel E. Quantification of Interplay Effects of Scanned Particle Beams and Moving Targets. Phys Med Biol (2008) 53(9):2253–65. doi: 10.1088/0031-9155/53/9/003 18401063

[3] Bert C Durante M. Motion in Radiotherapy: Particle Therapy. Phys Med Biol (2011) 56(16):R113–44. doi: 10.1088/0031-9155/56/16/R01 21775795

[4] Michelle L Wayne N Marco D Moritz W Timo S Athena P. Dosimetric Validation of a System to Treat Moving Tumors Using Scanned Ion Beams That Are Synchronized With Anatomical Motion. Front Oncol (2021) 11:712126. doi: 10.3389/fonc.2021.712126 34568041 PMC8456027

[5] Graeff C. Motion Mitigation in Scanned Ion Beam Therapy Through 4D-Optimization. Phys Med (2014) 30(5):570–7. doi: 10.1016/j.ejmp.2014.03.011 24818997

[6] Eley JG Newhauser WD Luchtenborg R Graeff C Bert C. 4D Optimization of Scanned Ion Beam Tracking Therapy for Moving Tumors. Phys Med Biol (2014) 59(13):3431–52. doi: 10.1088/0031-9155/59/13/3431 PMC4139294 24889215

[7] Minohara S Kanai T Endo M Noda K Kanazawa M. Respiratory Gated Irradiation System for Heavy-Ion Radiotherapy. Int J Radiat Oncol Biol Phys (2000) 47(4):1097–103. doi: 10.1016/S0360-3016(00)00524-1 10863083

[8] Kubo HD Hill BC. Respiration Gated Radiotherapy Treatment: A Technical Study. Phys Med Biol (1996) 41(1):83–91. doi: 10.1088/0031-9155/41/1/007 8685260

[9] Furukawa T Inaniwa T Sato S Shirai T Mori S Takeshita E. Moving Target Irradiation With Fast Rescanning and Gating in Particle Therapy. Med Phys (2010) 37(9):4874–9. doi: 10.1118/1.3481512 20964205

[10] Zenklusen SM Pedroni E Meer D. A Study on Repainting Strategies for Treating Moderately Moving Targets With Proton Pencil Beam Scanning at the New Gantry 2 at PSI. Phys Med Biol (2010) 55(17):5103–21. doi: 10.1088/0031-9155/55/17/014 20702927

[11] Mori S Furukawa T Inaniwa T Zenklusen S Nakao M Shirai T. System Evaluation of Four-Dimensional Hybrid Depth Scanning for Carbon-Ion Lung Therapy. Med Phys (2013) 40(3):031720. doi: 10.1118/1.4792295 23464315

[12] Takahashi W Mori S Nakajima M Yamamoto N Inaniwa T Furukawa T. Carbon-Ion Scanning Lung Treatment Planning With Respiratory-Gated Phase-Controlled Rescanning: Simulation Study Using 4-Dimensional CT Data. Radiat Oncol (2014) 9:238. doi: 10.1186/s13014-014-0238-y 25384996 PMC4230758

[13] Zhang Y Knopf AC Weber DC Lomax AJ. Improving 4D Plan Quality for PBS-Based Liver Tumor Treatments by Combining Online Image Guided Beam Gating With Rescanning. Phys Med Biol (2015) 60(20):8141–59. doi: 10.1088/0031-9155/60/20/8141 26439493

[14] Furukawa T Inaniwa T Sato S Tomitani T Minohara S Noda K. Design Study of a Raster Scanning System for Moving Target Irradiation in Heavy-Ion Radiotherapy. Med Phys (2007) 34(3):1085–97. doi: 10.1118/1.2558213 17441254

[15] Tsunashima Y Vedam S Dong L Umezawa M Sakae T Bues M. Efficiency of Respiratory-Gated Delivery of Synchrotron-Based Pulsed Proton Irradiation. Phys Med Biol (2008) 53(7):1947–59. doi: 10.1088/0031-9155/53/7/010 18364549

[16] Tsunashima Y Vedam S Dong L Umezawa M Balter P Mohan R. The Precision of Respiratory-Gated Delivery of Synchrotron-Based Pulsed Beam Proton Therapy. Phys Med Biol (2010) 55(24):7633–47. doi: 10.1088/0031-9155/55/24/016 21113089

[17] Iwata Y Furukawa T Noda K Shirai T Uchiyama H. Update of an Accelerator Control System for the New Treatment Facility at HIMAC. Proc EPAC08 (2008).

[18] Iwata Y Kadowaki T Uchiyama H Fujimoto T Takada E Shirai T. Multiple-Energy Operation With Extended Flattops at HIMAC. Nucl Instrum Methods A (2010) 624:33–8. doi: 10.1016/j.nima.2010.09.016

[19] Furukawa T Hara Y Mizushima K Saotome N Tansho R Saraya Y. Development of NIRS Pencil Beam Scanning System for Carbon Ion Radiotherapy. Nucl Instrum Methods B (2016) 406:361–7. doi: 10.1016/j.nimb.2016.10.029

[20] Mizushima K Furukawa T Hara Y Hara Y Saotome N Saraya Y. Performance of the HIMAC Beam Control System Using Multiple-Energy Synchrotron Operation. Nucl Instrum Methods B (2017) 406:347–51. doi: 10.1016/j.nimb.2017.03.051

[21] Mori S Inaniwa T Furukawa T Takahashi W Nakajima M Shirai T. Amplitude-Based Gated Phase-Controlled Rescanning in Carbon-Ion Scanning Beam Treatment Planning Under Irregular Breathing Conditions Using Lung and Liver 4dcts. J Radiat Res (2014) 55(5):948–58. doi: 10.1093/jrr/rru032 PMC4202290 24835238

[22] He P Li Q Liu X Dai Z Zhao T Fu T. Respiratory Motion Management Using Audio-Visual Biofeedback for Respiratory-Gated Radiotherapy of Synchrotron-Based Pulsed Heavy-Ion Beam Delivery. Med Phys (2014) 41(11):111708. doi: 10.1118/1.4897391 25370622 PMC4218689

[23] He P Li Q Zhao T Liu X Dai Z Ma Y. Effectiveness of Respiratory-Gated Radiotherapy With Audio-Visual Biofeedback for Synchrotron-Based Scanned Heavy-Ion Beam Delivery. Phys Med Biol (2016) 61(24):8541–52. doi: 10.1088/0031-9155/61/24/8541 27845937

[24] Zhang Y Huth I Weber DC Lomax AJ. A Statistical Comparison of Motion Mitigation Performances and Robustness of Various Pencil Beam Scanning Proton Systems for Liver Tumour Treatments. Radiother Oncol (2018) 128(1):182–8. doi: 10.1016/j.radonc.2018.01.019 29459153

[25] Graeff C Luchtenborg R Eley JG Durante M Bert C. A 4D-Optimization Concept for Scanned Ion Beam Therapy. Radiother Oncol (2013) 109(3):419–24. doi: 10.1016/j.radonc.2013.09.018 24183865

[26] Shackleford JA Kandasamy N Sharp GC. On Developing B-Spline Registration Algorithms for Multi-Core Processors. Phys Med Biol (2010) 55(21):6329–51. doi: 10.1088/0031-9155/55/21/001 20938071

[27] Knopf AC Boye D Lomax A Mori S. Adequate Margin Definition for Scanned Particle Therapy in the Incidence of Intrafractional Motion. Phys Med Biol (2013) 58(17):6079–94. doi: 10.1088/0031-9155/58/17/6079 23939146

[28] Wieser HP Cisternas E Wahl N Ulrich S Stadler A Mescher H. Development of the Open-Source Dose Calculation and Optimization Toolkit Matrad. Med Phys (2017) 44(6):2556–68. doi: 10.1002/mp.12251 28370020

[29] He P Li Q. Motion Management With Variable Cycle-Based Respiratory Guidance Method for Carbon-Ion Pencil Beam Scanning Treatment. Physica Med (2021) 87:99–105. doi: 10.1016/j.ejmp.2021.06.005 34134014

[30] Zhang Y Huth I Weber DC Lomax AJ. Dosimetric Uncertainties as a Result of Temporal Resolution in 4D Dose Calculations for PBS Proton Therapy. Phys Med Biol (2019) 64(12):125005. doi: 10.1088/1361-6560/ab1d6f 31035271

[31] Shen L Zhao W Xing L. Patient-Specific Reconstruction of Volumetric Computed Tomography Images From a Single Projection View *via* Deep Learning. Nat BioMed Eng (2020) 3(11):880–8. doi: 10.1038/s41551-019-0466-4 PMC6858583 31659306

